# Comparative Genomic Analysis of Asian Haemorrhagic Septicaemia-Associated Strains of *Pasteurella multocida* Identifies More than 90 Haemorrhagic Septicaemia-Specific Genes

**DOI:** 10.1371/journal.pone.0130296

**Published:** 2015-07-07

**Authors:** Ahmed M. Moustafa, Torsten Seemann, Simon Gladman, Ben Adler, Marina Harper, John D. Boyce, Mark D. Bennett

**Affiliations:** 1 School of Veterinary and Life Sciences, Murdoch University, South Street, Perth, Western Australia, Australia; 2 Victorian Bioinformatics Consortium, Monash University, Wellington Road, Clayton, Melbourne, Victoria, Australia; 3 Australian Research Council Centre of Excellence in Structural and Functional Microbial Genomics, Monash University, Wellington Road, Clayton, Melbourne, Victoria, Australia; 4 Department of Microbiology, Monash University, Wellington Road, Clayton, Melbourne, Victoria, Australia; 5 Victorian Life Sciences Computation Initiative, Grattan Street, Carlton, Melbourne, Victoria, Australia; Beijing Institute of Genomics, CHINA

## Abstract

*Pasteurella multocida* is the primary causative agent of a range of economically important diseases in animals, including haemorrhagic septicaemia (HS), a rapidly fatal disease of ungulates. There is limited information available on the diversity of *P*. *multocida* strains that cause HS. Therefore, we determined draft genome sequences of ten disease-causing isolates and two vaccine strains and compared these genomes using a range of bioinformatic analyses. The draft genomes of the 12 HS strains were between 2,298,035 and 2,410,300 bp in length. Comparison of these genomes with the North American HS strain, M1404, and other available *P*. *multocida* genomes (Pm70, 3480, 36950 and HN06) identified a core set of 1,824 genes. A set of 96 genes was present in all HS isolates and vaccine strains examined in this study, but absent from Pm70, 3480, 36950 and HN06. Moreover, 59 genes were shared only by the Asian B:2 strains. In two Pakistani isolates, genes with high similarity to genes in the integrative and conjugative element, ICE*Pmu1* from strain 36950 were identified along with a range of other antimicrobial resistance genes. Phylogenetic analysis indicated that the HS strains formed clades based on their country of isolation. Future analysis of the 96 genes unique to the HS isolates will aid the identification of HS-specific virulence attributes and facilitate the development of disease-specific diagnostic tests.

## Introduction

*Pasteurella multocida* is a Gram-negative, nonmotile, nonspore-forming coccobacillus. It is the causative agent of a spectrum of economically important diseases worldwide, including atrophic rhinitis in pigs, haemorrhagic septicaemia (HS) in cattle and buffalo, fowl cholera in poultry, snuffles in rabbits and sporadic human infections that often follow dog or cat bites [1,2]. *P*. *multocida* is a heterogeneous species with strains being commonly differentiated by serology [3], or more recently capsular locus-specific multiplex PCR [4], into five capsular serogroups designated A, B, D, E and F. Strains belonging to capsular serogroups A, D and F produce capsules composed of hyaluronic acid, heparin and chondroitin respectively [4]. The composition of the B and E capsules is unknown but the genes required for their biosynthesis have been defined [4]. A second serological typing system is also often used to differentiate strains into 16 Heddleston serotypes or serovars based on lipopolysaccharide (LPS) antigens [5]. Full strain designations usually combine both systems, such that a designation of B:2 indicates capsule serogroup B and LPS serovar 2.

Haemorrhagic septicaemia (HS) is an acute and generally fatal disease which occurs mainly in cattle and buffalo [2]. Haemorrhagic septicaemia is prevalent in Asia and Africa, where its presence is of great economic importance. In Pakistan, it has been reported as the most economically important bacterial disease of cattle and buffalo [6]. Similarly, in Thailand, HS ranks high on the list of economically important diseases of livestock [7]. Haemorrhagic septicaemia is caused by infection with *P*. *multocida* strains belonging to capsular serogroups B and E [2]. Haemorrhagic septicaemia strains producing a serogroup B capsule are predominant in Asia while strains producing a serogroup E capsule are predominant in Africa, although African serogroup B isolates and Asian serogroup E isolates have occasionally been reported [8]. *P*. *multocida* strains that cause HS belong to the LPS serovars 2 or 5 which share the same LPS outer core biosynthesis locus and produce structurally highly related, but antigenically distinct, LPS molecules [9].

The first complete genome sequence of a *P*. *multocida* strain (Pm70; GenBank accession AE004439) was determined in 2001 [10]. Analysis of the Pm70 genome identified more than 100 genes predicted to be involved in virulence and identified complete gene sets for the following pathways: TCA cycle, glycolysis, glyconeogenesis, oxidative pentose phosphate and Entner—Doudoroff [10].

There are currently 25 publicly available complete or draft *P*. *multocida* genomes. These genomes are from strains isolated from different hosts and which cause a range of different diseases [11]. There have been only limited comparative analyses of these different genomes. However, analysis of the Pm70, 36950, 3480, HN06, X73, and P1059 genomes identified a unique 18 kbp region in the porcine atrophic rhinitis isolate, HN06, that contained 14 genes, including the *toxA* gene encoding the *P*. *multocida* toxin (PMT) (which causes the signs of atrophic rhinitis) as well as several phage-related genes [12]. Further analyses also showed that an integrative conjugative element (ICE), ICE*Pmu1*, was found in bovine respiratory disease isolate 36950, but not in any of the other strains. This element carried 11 different antibiotic resistance genes. A similar ICE has also been found in *Histophilus somni* and *Mannheimia haemolytica* which are both bovine respiratory pathogens [12].

Previous analysis of five *P*. *multocida* genomes (M1404, Pm70, 36950, X73, and P903) identified a core set of 1786 genes (88% of Pm70 gene content) common to all strains and a pan genome of more than 2,800 genes. Furthermore, each of these strains contained between 90 and 261 unique genes not found in any of the other strains examined [13]. For strain 36950, more than 47% of the unique genes identified were within the ICE*Pmu1* element, whereas for strain M1404 28% of unique genes were phage-derived elements. Importantly, a previous phylogenetic comparison of nine *P*. *multocida* strains indicated little correlation between phylogenetic relationship and disease type, capsular/LPS type, host predilection or place of isolation [13].

We recently analyzed the genotypes of 23 *P*. *multocida* isolates, 14 recovered from HS-diseased cattle and buffalo located in different geographical areas and climate zones of Pakistan and nine from different regions of Thailand. All isolates were serovar B:2 and indistinguishable by multi locus sequence typing (MLST) with all strains sequence type 122. Furthermore, all isolates from within each country were indistinguishable by pulsed-field gel electrophoresis (PFGE) [14]. Therefore, to determine whether there was any diversity across these isolates we determined whole draft genome sequences of a selection of 12 of the strains using next generation sequencing (NGS). The draft genomes were then compared with the M1404 genome (a North American serovar B:2 HS-associated strain) and the genomes from four strains not associated with HS (Pm70, 36950, 3480 and HN06). To our knowledge, this is the first detailed genomic analysis of multiple HS-associated isolates of *P*. *multocida*.

## Materials and Methods

Ten *P*. *multocida* strains that had been collected from buffalo or cattle with HS, each from different regions of Pakistan or Thailand, were sourced from the National Veterinary Laboratory in Islamabad or the Department of Livestock Development in Thailand, respectively. In addition, two vaccine strains from Pakistan were also included in this study (Table 1). Except for the Faisalabad isolate (Table 1), all of the isolates had previously been identified as *P*. *multocida* and typed using MLST (all sequence type 122) [14]. The Faisalabad isolate was received from the National Veterinary Laboratory, Islamabad, Pakistan, and identified as a HS-associated *P*. *multocida* strain using both a *P*. *multocida*-specific and a HS-specific PCR (data not shown) [15].

**Table 1:** Haemorrhagic septicaemia-associated strains of *Pasteurella multocida* used in this study.

Isolate	Strain abbreviation	Host	Year	Country	Province	District	Location coordinates
Thailand A	THA	Buffalo	2006	Thailand	Nakhon Si Thammarat	Thung song	8.16N, 99.68E
Thailand D	THD	Buffalo	2009	Thailand	Chonburi	Phanat Nikhom	13.45N, 101.18E
Thailand F	THF	Buffalo	2011	Thailand	Lamphun	Mueang Lamphun	18.58N, 99.02E
Attock	ATTK	Cattle	2010	Pakistan	Punjab	Attock	33.91N, 72.31E
Bhakkar	BUKK	Cattle	2008	Pakistan	Punjab	Bhakkar	31.63N, 71.07E
Taxila 1	TX1	Buffalo	2012	Pakistan	Punjab	Rawalpindi	33.75N, 72.79E
Karachi 3	Karachi	Buffalo	2011	Pakistan	Sindh	Karachi	24.86N, 67.01E
Islamabad 1	Islm	Wild Buffalo	2011	Pakistan	Islamabad capital territory	Islamabad	33.72N, 73.07E
Peshawar	Pesh	Buffalo	2011	Pakistan	Khyber Pakhtunkhwa	Peshawar	34.02N, 71.58E
Peshawar vaccine	PVAcc	NA	2011	Pakistan	Khyber Pakhtunkhwa	Peshawar	34.02N, 71.58E
Lahore Vaccine	V1	Buffalo	2011	Pakistan	Punjab	Lahore	31.55N, 74.34E
Faisalabad	Faisal	Buffalo	2011	Pakistan	Punjab	Faisalabad	31.418N, 73.079E

### Ethics statement

All strains were sourced from the National Veterinary Laboratory in Islamabad or the Department of Livestock Development in Thailand so no ethics approvals were required.

### Whole genome sequencing

Genomic DNA was purified from each of the twelve *P*. *multocida* strains using the Qiagen DNeasy blood and tissue kit (Qiagen Cat# 69504) using 5 mL of overnight cultures grown at 37°C in brain heart infusion (BHI) broth (Oxoid, UK) and following the Gram-negative bacterial protocol outlined in the manufacturer’s instructions. DNA quantification and purity analysis was performed by agarose gel electrophoresis and Qubit Fluorometry (Life Technologies, USA). The purified genomic DNA was sequenced using the paired-end 90-bp sequencing protocol on an Illumina HiSeq 2000 (Illumina Inc., San Diego, USA) at the Beijing Genomics Institute (BGI), China. The raw read sequences were filtered to eliminate low quality reads using the following criteria; all reads with > 40% low quality (Q20) bases (parameter setting at 36 bp), > 10% Ns (parameter setting at 9 bp) or > 15 bp overlap with Illumina TruSeq adapter sequences (parameter setting at 15 bp) were removed.

### Sequence assembly

The genomes of ten of the strains were *de novo* assembled using SPAdes v2.5.0 [16] and the remaining two strains were assembled using Velvet v1.2.07 [17]. For ten of the twelve strains, the SPAdes procedure generated acceptable assemblies. However, for the Faisal and ATTK strains, SPAdes produced unexpectedly large genome assemblies (8,278,703 and 6,134,337 bp for Faisal and ATTK, respectively) due to low level contamination with genomic DNA from another bacterial species (*Bacillus cereus* and *Bacillus subtilis*, respectively). Examination of the Velvet statistics showed that the contaminating sequences were represented by short contigs having sequencing depth below 10x. To filter this contamination, we assembled the Faisal and ATTK sequences using Velvet with a manual setting of 10 for the minimum required coverage (“velvetg-cov_cutoff 10”) to remove these undesirable components of the assembly graph prior to repeat resolution and contig extraction. Final genome assemblies of these strains were also checked for contaminating sequences using BLASTN v2.2.26 [18,19]. To evaluate the accuracy of the generated contigs of the 12 assembled genomes, they were compared with the Pm70 reference genome [10] using QUAST v2.3 [20]; sequence and assembly statistics of the 12 genomes are given in Table 2. For all strains, scaffolds (or contigs for the Velvet assembled genomes) of less than 200 bp in length were removed before the final reordering using Mauve [21] with Pm70 used as the reference sequence. The final ordered and oriented scaffold sequences were then annotated using the NCBI Prokaryotic Genome Annotation Pipeline [22]. The 12 annotated genomes were submitted to GenBank [23,24]; accession numbers are given in Table 3.

**Table 2:** Sequencing and assembly statistics for the genomes of the 12 Asian HS-associated strains.

Strain	Sequence yield Mb)	Number of contigs	Largest contig (bp)	N50	N75	Sequence coverage^1^ (%)	Average sequence depth
ATTK	251	44	373941	265021	106344	92.17	96
BUKK	251	42	589995	289440	106424	91.95	110
Faisal	250	52	374481	265074	106436	92.15	68
Karachi	251	77	647631	289467	111128	92.17	100
Islm	252	35	647631	289467	111128	92.17	100
Pesh	250	35	647631	289467	111128	92.17	100
PVAcc	252	41	647631	289467	111128	92.17	100
THA	251	33	594579	289958	111130	92.19	100
THD	252	35	594578	289367	111130	92.19	100
THF	250	32	549574	290056	111130	92.18	100
TX1	250	40	635052	289440	106424	92.16	100
V1	251	32	647631	289395	111128	92.17	100

^1^Sequence coverage of the 12 assembled genomes is given relative to Pm70 (reference genome).

**Table 3:** Genomic features of the 12 Asian strains.

Strain	Genome Size (kbp)	Number of CDS	tRNA	rRNA	Accession number
ATTK	2399	2141	47	10^1^	JQEA00000000
BUKK	2410	2148	50	4	JQAO00000000
Faisal	2405	2143	52	13^1^	JQEB00000000
Karachi	2419	2164	51	5	JPHI00000000
Islm	2396	2142	51	4	JQAB00000000
Pesh	2397	2144	51	4	JQAC00000000
PVAcc	2399	2140	50	4	JQAD00000000
THA	2344	2082	50	4	JQAE00000000
THD	2344	2082	50	4	JQAF00000000
THF	2344	2082	51	4	JQAG00000000
TX1	2458	2179	50	4	JQAH00000000
V1	2396	2137	50	4	JQAI00000000

^1^ The high number of rRNA operons in these strains is due to contig breaks

### Variant calls: single-nucleotide polymorphisms (SNPs) and insertion/deletion polymorphisms (indels)

Snippy (available at https://github.com/tseemann/snippy) was used to identify SNPs and Indels in the NGS sequence reads (FASTQ format) from each of the 12 genomes compared with the reference genome, Pm70.

### Genome alignments and feature analysis

Mauve v2.3.1 (default settings) [25] was used to align the genomes of the twelve Pakistani and Thai *P*. *multocida* strains with the genomes of the following strains; M1404 (bovine HS isolate, type B:2) [13], Pm70 (avian fowl cholera isolate) [10], HN06 (porcine atrophic rhinitis isolate) [26] and 3480 (GenBank: NC_017764.1) and 36950 [27] (porcine and bovine respiratory disease isolate respectively). The “homologs table” in Mauve was used to identify colinear orthologs across each of the genomes using 50% DNA identity and 50% gene coverage as the minimum criteria for a match. All genes present only in a single strain were then manually checked by BLAST [18,19] to confirm that they were unique. Genes unique to the Pakistani or Thai strains, or unique to M1404, were also identified by this method.

The PHAge Search Tool (PHAST) [28] was used to identify the positions of putative phage elements in all genomes. For PHAST analysis the FASTA files containing all concatenated contigs for each genome were uploaded to the public PHAST web server (http://phast.wishartlab.com/). To avoid false positives caused by non phage-related mobile genetic elements, PHAST filters out these mobile genetic elements using a two-step process. In the first step, PHAST uses a customized mobile genetic element database to directly filter out some of the most typical mobile genetic elements (Y. Zhou, personal communication). In the second step, PHAST filters out the rest of the mobile genetic elements when it identifies potential prophages by the density-based spatial clustering of applications with noise (DBSCAN) algorithm [28]. Specifically, the DBSCAN algorithm marks out the random mobile genetic elements as noise and clusters other gene elements into potential prophages (Y. Zhou, personal communication). In addition, PHAST predicts potential prophages based on a number of factors including the relative density of identified prophage-like genes, GC ratio, functional completeness and gene similarity to already known phages. The genomes were also checked for the presence of antimicrobial resistance genes using the ResFinder tool [29].

The capsule biosynthesis and LPS outer core biosynthetic loci were identified by BLAST comparison [18,19] against the previously reported M1404 gene clusters for capsule (20,418 bp in size) and LPS outer core (4,887 bp in size) biosynthesis. The presence and integrity of the two LPS heptosyltransferase genes, *hptA* and *hptB* were checked in order to predict whether the two different, simultaneously expressed, LPS inner core structures (glycoform A and glycoform B) were present [30]. In addition, the twelve strains were classified into either Heddleston serovar 2 or 5 by analysis of the *lpt-3* gene, required for the addition of phosphoethanolamine (PEtn) to the 3 position of the second heptose (Hep II) [9].

### Phylogenetic trees

The phylogenetic relationship between the strains was predicted by analysis of core genome single nucleotide polymorphisms (CG-SNPs). Identification of CG-SNPs and phylogenetic analysis was assessed using Wombac v1.2 (https://github.com/tseemann/wombac). The four closely related species, *Gallibacterium anatis*, *Mannheimia haemolytica*, *P*. *bettyae* and *P*. *dagmatis* were used as outgroups. The *P*. *multocida* strains used in these analyses were the known HS-associated strains VTCCBAA264 (GenBank: ALYC00000000) [31], P52VAC (GenBank: ALBZ00000000), Anand1C (GenBank: ALBY00000000), Anand1B (GenBank: ALBX00000000), M1404 [13] and the 12 HS isolates sequenced in this study (Table 1); the fowl cholera isolates Pm70 (GenBank: NC_002663.1), X73, (GenBank: AMBP00000000) [32], VP161 [13], P1059 (GenBank: AMBQ00000000) [32] and Anand1P (GenBank: AFRR00000000) [33]; the porcine lung isolates HN06, (GenBank: NC_017027.1) [26] and 3480, (GenBank: NC_017764.1); the bovine respiratory disease isolate 36950, (Genbank: NC_016808.1) [27] and the caprine isolate Anand1G, (GenBank: AFRS00000000). The uncorrected *p* method, which computes the proportion of positions at which two sequences differ, was used to build the distance matrix and determine distances between different strains. Phylogenetic trees were constructed with SplitsTree v4.11.3 [34] using the neighbour-joining method and uncorrected *p* distance matrices.

## Results and Discussion

### Genome sequencing of 12 *P*. *multocida* HS strains

Ten *P*. *multocida* strains, isolated from HS cases in buffalo (8 isolates) and cattle (2 isolates) from Pakistan and Thailand, and two Pakistani HS vaccine strains were used for this study (Table 1). Except for the Faisalabad isolate (Table 1), all of the isolates had previously been genotyped using MLST as ST122 [14]. Therefore, in order to identify if there were differences between the strains, we determined whole genome draft sequences of each strain. Genomic DNA was isolated from each strain and sequenced on an Illumina HiSeq 2000. Sequences reads were *de novo* assembled, resulting in between 32 (strains THF and V1) and 77 (strain Karachi) contigs of > 200 bp. The genomic features of the 12 sequenced strains and accession numbers are shown in Table 3. The predicted genome sizes ranged from 2.34 to 2.46 Mbp and the number of coding sequences (CDS) ranged between 2,082 (strains THA, THD and THF) and 2,179 (strain TX1). The GC content was highly conserved across all strains (40.31 to 40.41%).

### Variant calls and genetics of capsule and LPS biosynthesis in the HS-causing strains

Mapping of the sequencing reads of the 12 Asian genomes to the complete genome of Pm70 identified between 16,443 and 16,513 CDS SNPs in the 12 genomes (Table 4). Furthermore, between 12 and 19 indels were also identified in the CDS of the 12 genomes (Table 4).

**Table 4:** Single-nucleotide polymorphisms (SNPs) and insertion/deletion polymorphisms (indels) in the twelve Asian genomes.

Strain	Indels (intergenic)	Indels (CDS)	SNPs (intergenic)	SNPs (CDS)
ATTK	99	18	1630	16503
BUKK	98	17	1619	16443
Faisal	102	17	1612	16471
Karachi	101	19	1612	16492
Islm	109	15	1608	16486
Pesh	91	14	1600	16491
PVAcc	103	12	1606	16492
THA	96	15	1625	16494
THD	98	19	1617	16513
THF	90	15	1613	16484
TX1	96	19	1619	16512
V1	98	17	1616	16491

All HS-associated strains contained the type B capsular biosynthetic locus. Only three nucleotide changes were observed in the *cap* locus of all of the Asian strains compared to the *cap* locus of M1404 [35]. Two of the mutations were silent, while the third encoded a missense mutation (D50Y) within the putative glycosyltransferase EpsJ. An additional nucleotide change in a non-coding region was observed in the Pakistani strains (relative position: 1185274 in BUKK). The FASTA file of the *cap* locus of strain BUKK is provided as S1 File.

All HS-associated strains also contained the LPS outer core biosynthesis locus that is shared by strains belonging to Heddleston serovars 2 and 5 [9]. Only a single nucleotide change was observed across the LPS outer core biosynthesis loci of the 12 Asian strains when compared with the M1404 locus [9]. The FASTA file of the LPS biosynthesis locus of strain BUKK is provided as S2 File. The Heddleston 2 and 5 type strains can be differentiated serologically by the presence (serovar 5) or absence (serovar 2) of PEtn on the 2^nd^ inner core heptose of the LPS [9]. Addition of this PEtn residue to the LPS is dependent on the presence of an intact *lpt-3* gene (annotated as *dcaA* in Pm70) [9]. All HS strains contained a disrupted *lpt-3* gene, with a nonsense mutation (relative position: 499751 in BUKK strain) identical to the mutation previously reported in M1404 [9]. Therefore, all of the HS strains analysed in this study are predicted to belong to LPS serovar 2. Indeed, all of the Pakistani and Thai strains had previously been classified using Heddleston serology as LPS serovar 2, except for THD which had been reported as LPS serovar 2,5 (P. Pathanasophon, personal communication, June, 2012). However, our genetic analyses would indicate that THD also belongs to LPS serovar 2, highlighting the advantage of molecular over serological typing methods.

It has been shown previously that most *P*. *multocida* strains produce two inner core LPS glycoforms designated A and B [30]. Production of these two glycoforms is dependent on the presence of two active heptosyltransferases, HptA and HptB. HptA is specific for inner core glycoform A and is responsible for the addition of the first heptose to a single phosphorylated 3-deoxy-D-manno-octulosonic acid (Kdo). For glycoform B assembly, unphosphorylated Kdo residues have a second Kdo residue added followed by the addition of the first heptose by a different heptosyltransferase, HptB [30]. The *hptB* gene in all strains included in this study was intact, indicating that HptB would be fully functional. The *hptA* gene was intact in all strains included in this study except for strain 3480 where it contained a premature stop codon (mutation at 2232375). Therefore, we would predict that strain 3480 is unable to add the first heptose to the glycoform A inner core. Interestingly, a *hptA* mutant constructed in the *P*. *multocida* fowl cholera isolate VP161 was significantly attenuated for virulence, predicted to be due to the presence of truncated glycoform A LPS on the surface of the cell [30]. However, in a further study it was found that growth of the *hptA* mutants *in vivo* selected for virulent strains with nonsense suppressor mutations in the Kdo phosphokinase gene, *kdtA*. This mutation prevented the phosphorylation of any Kdo residues, allowing all Kdo residues to be available for glycoform B LPS assembly [36].

### Phylogeny of the tested strains

**Fig 1:**
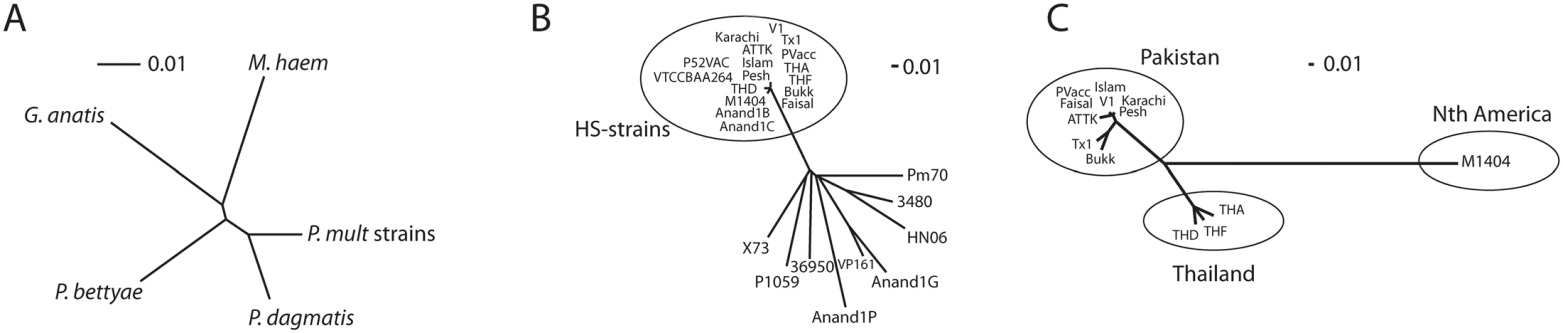
Unrooted neighbour-joining trees showing the phylogenetic relationship between various strains. A. Relationship between *Gallibacterium anatis*, *Mannheimia haemolytica*, *Pasteurella dagmatis*, *Pasteurella bettyae* and the *P*. *multocida* strains Pm70, 36950, HN06, P3480, X73, VP161, Anand1C, Anand1B, Anand1P, Anand1G, P1059, P52VAC, VTCCBAA264, M1404 and the twelve Pakistani and Thai isolates. B. Relationship between the *P*. *multocida* strains. C. Relationship between the HS-associated *P*. *multocida* B:2 strain M1404 and the twelve Pakistani and Thai isolates. Phylogenetic relatedness for all comparisons was determined by analysis of only the single nucleotide polymorphisms found at conserved positions in all genomes of the comparison set (CG-SNPs); 789 shared positions for the tree in panel A, 7,829 shared positions for the tree in panel B and 722 shared positions for the tree in panel C. Trees were rendered with SplitsTree v4.11.3 [34]. The line segments above the trees with the number '0.01' indicate the branch length representing a genetic change of 0.01.

The relatedness of the different strains was determined by comparing all nucleotide changes at positions that were conserved across all of the comparison genomes (core genome single nucleotide polymorphisms; CG-SNPs). Firstly, all the *P*. *multocida* strains were compared together with the closely related species *P*. *dagmatis*, *P*. *bettyae*, *Gallibacterium anatis* and *Mannheimia haemolytica*. This analysis, using 789 CG-SNPs, clearly showed that all the *P*. *multocida* strains form a monophyletic group most closely related to *P*. *dagmatis* (Fig 1A). Secondly, comparison of only *P*. *multocida* strains using 7,892 CG-SNPs (Fig 1B), indicated that the *P*. *multocida* HS strains were very closely related and clearly separated from all of the other *P*. *multocida* strains. This finding is in contrast to previous analyses using a smaller number of strains that showed little or no correlation between phylogeny and serovar, disease type or host predilection [13]. However, there was still no clear correlation between strain relatedness and disease type other than for the HS strains. Indeed, the five fowl cholera isolates (Pm70, X73, VP161, P1059 and Anand1P) did not cluster separately from strains associated with other disease types. Finally, we compared just the HS strains sequenced in this study and M1404 using 722 CG-SNPs (Fig 1C). There was a clear separation between strain M1404 (the North American isolate) and the Thai and Pakistani strains, which were also clearly separated from each other. Therefore, the higher resolution provided by whole genome sequencing revealed a clear genetic relationship with geographic source which was not possible with MLST.

### Core and pan genome predictions

**Fig 2:**
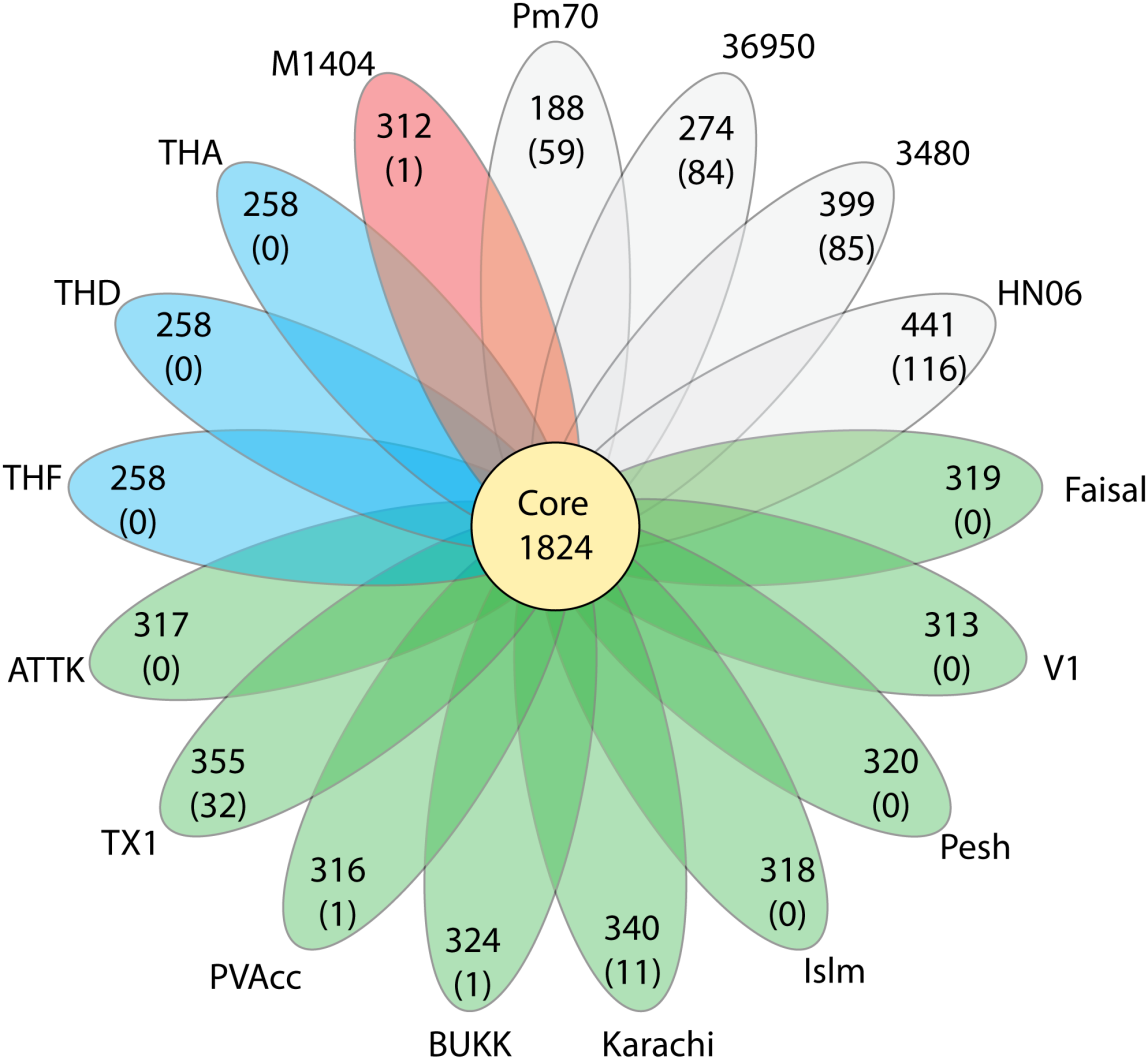
Flower plot diagram showing core and unique genes across all strains. The central circle shows the number of genes common to all strains while the petals show the number of genes in addition to the core set, as well as the number of genes unique to each strain (in brackets). Abbreviated strain names are given outside each petal, strain details are given in Table 1. The B:2 HS-related strains are shaded as follows; M1404 is orange, Thai strains are blue and the Pakistani strains are green. Non-B:2 strains are shaded in grey.

We analysed the gene content of each of the 12 sequenced HS strains and compared these predictions with the coding sequences predicted for the four complete and annotated *P*. *multocida* genomes (36950, Pm70, 3480, HN06) and the M1404 draft genome. These analyses identified a shared set of 1,824 genes (core) and a pan genome of more than 2,700 genes (Fig 2). Furthermore, with the exception of the Thai isolates THA, THD and THF and the Pakistani isolates Pesh, Islm, V1, Faisal and ATTK, all other strains contained genes not found in any of the other strains used in the analysis (Fig 2). The unique genes in each of the four strains (BUKK, PVAcc, Karachi and Tx1) are provided in S1 Table. The genome from strains 36950 and 3480 contained 84 and 85 unique genes respectively; Pm70 contained 59 unique genes; strain TX1, 32 genes; strain Karachi, 11 genes and one unique gene was found in each genome of strains M1404, BUKK and PVAcc.

Functional comparison of the core genes and strain-specific genes showed that the core genes are mainly responsible for inorganic ion transport and metabolism, energy production and metabolism, cell membrane biogenesis, ribosomal biogenesis, amino acid transport and metabolism, coenzyme transport and metabolism, carbohydrate transport and metabolism, signal transduction, transcription, translation, replication and repair, lipid metabolism, membrane transport and hypothetical proteins. The two strain-specific genes for PVAcc and BUKK encode hypothetical proteins. The eleven unique genes for the Karachi strain encode eight putative transposases and three hypothetical proteins. For strain TX1, the 32 unique genes encode putative conjugal transfer proteins, a DNA topoisomerase III, transcriptional regulator proteins, proteins involved in DNA replication, a cobalt ABC transport system, helicase and an endonuclease.

**Fig 3:**
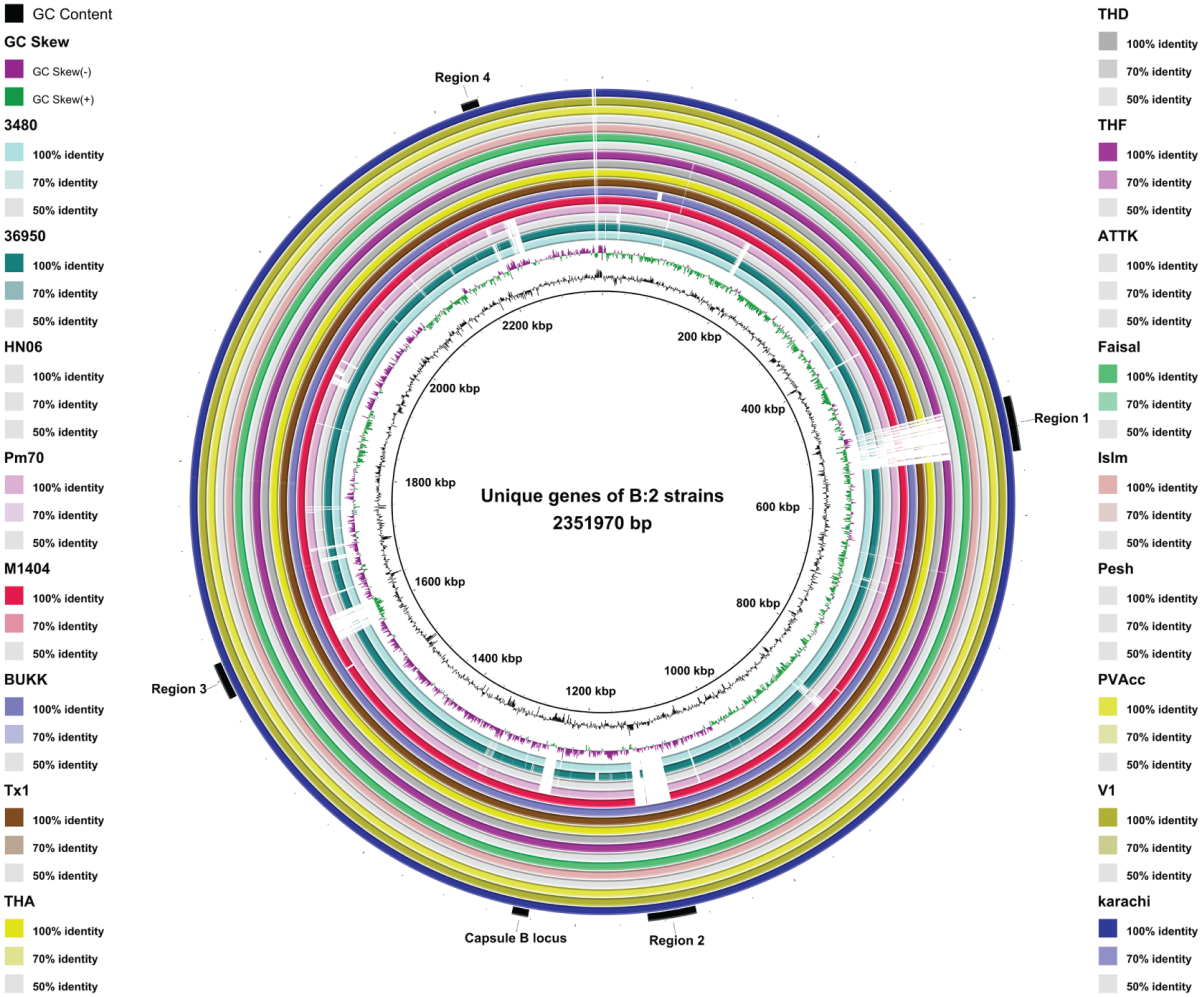
Comparison of the genomes of 3480, 36950, HN06, Pm70, M1404, BUKK, TX1, THA, THD, THF, ATTK, Faisal, Islm, Pesh, PVAcc, V1 and Karachi with the genome of the PVAcc strain. The three inner rings show the DNA size, GC content and GC skew of the reference genome (PVAcc strain). The 17 outer rings show regions of the comparison genomes that match the reference genome PVAcc and in the order (inside to outside) 3480, 36950, HN06, Pm70, M1404, BUKK, Tx1, THA, THD, THF, ATTK, Faisal, Islm, Pesh, PVAcc, V1 and Karachi. Regions 1–4 on the outside identify particular regions of difference between the strains that are potential prophage elements. The position of the type B capsule locus is also noted between regions 2 and 3. This figure was drawn using BLAST ring image generator (BRIG) [39].

**Fig 4:**
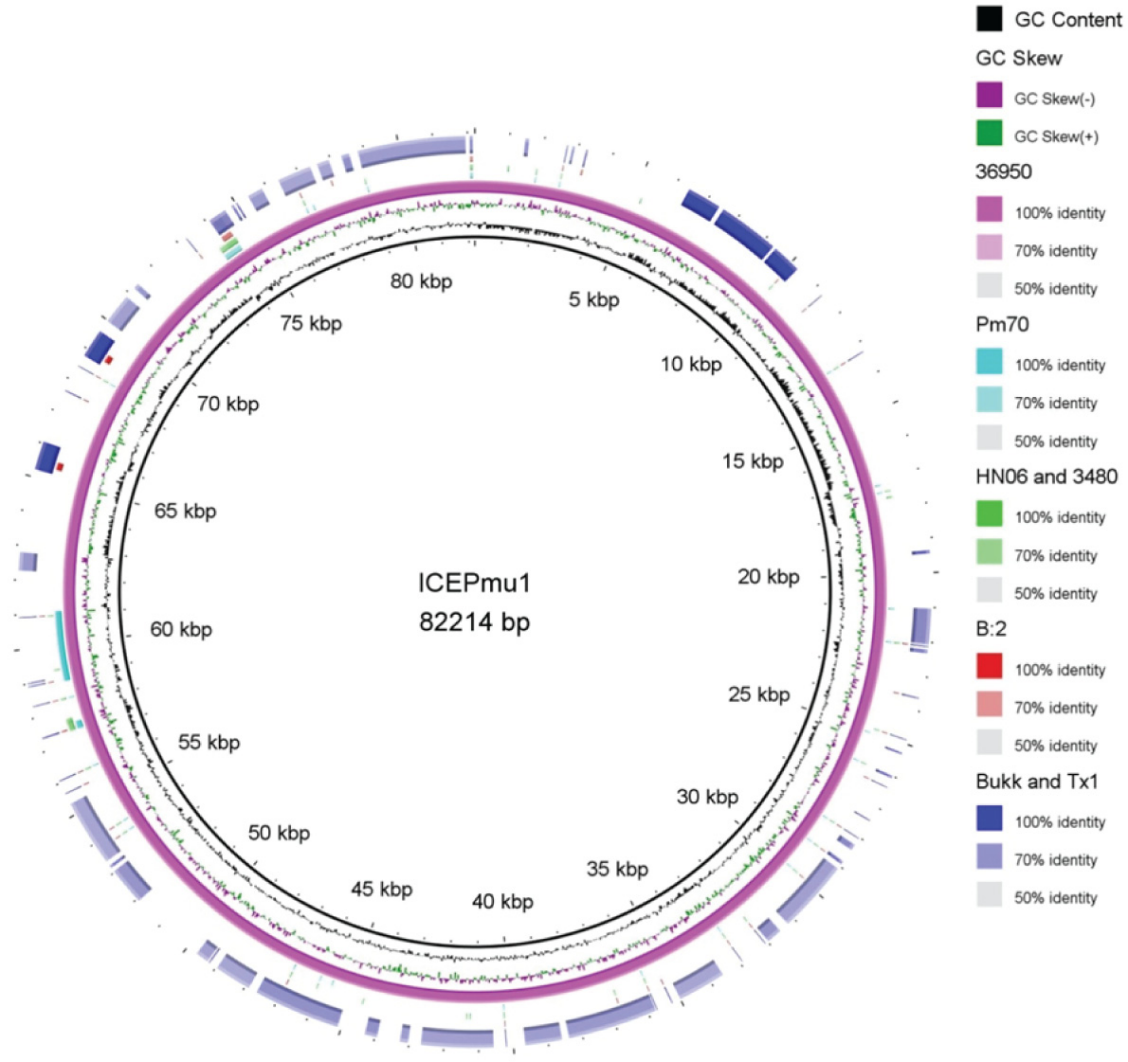
Comparison of the integrative conjugative element, ICE*Pmu1*, present in 36950 strain [33] with the genomes of 36950, Pm70, combined HN06 and 3480, combined M1404 (B:2), ATTK, BUKK, Faisal, Karachi, Islm, Pesh, PVAcc, THA, THD and THF strains and combined BUKK and TX1 strains, displayed as the outer rings inside to outside respectively. The three inner rings show the DNA size, GC content and GC skew of the reference element (ICE*Pmu1*). The five outer rings show regions of the comparison genomes that match the reference ICE*Pmu1*. Figure was drawn using BRIG [39].

These comparative analyses also showed that all HS-associated strains included in the pan-genome analysis (M1404, Pakistani and Thai strains) share two large regions of unique sequence compared to the other complete genomes. The first region is approximately 34 kb in length (region 3 in Fig 3) while the second region is approximately 15 kb in length (region 4 in Fig 3) (Table 5). Furthermore, there are several dispersed genes uniquely present in all of the HS strains. Overall the HS strains share 96 genes that are absent from the other genomes analysed, including the capsule biosynthesis locus, present in all strains belonging to capsular serogroup B (Fig 3). These genes unique to the HS strains are provided in S1 Table. In addition, the twelve Asian HS strains share an approximately 44 kb region (region 2 in Fig 3) (Table 5) that is absent from the American HS strain M1404; this region contains 59 genes that encode mostly proteins with no significant similarity to proteins of known function. The seven Pakistani strains (ATTK, Faisal, Islm, Karachi, Pesh, PVAcc and V1) share an approximately 50 kb region containing 39 genes that is not present in the other genomes (region 1 in Fig 3) (Table 5). Genes encoded in region 1 encode mostly phage elements as well as hypothetical proteins. Additionally, strains 36950, TX1 and BUKK share 35 unique genes, encoding elements with similarity to the integrative conjugative element (ICE*Pmu1*) of 36950 (Fig 4). There are also 42 genes shared by TX1 and BUKK. TX1 is the only Asian HS strain with a large number (32) of unique genes and these predominantly encode phage elements and hypothetical proteins. The three Thai HS isolates have just a single unique gene encoding an abortive infection-(Abi-) like protein of 226 amino acids. Abi-like genes are found in various bacterial species, and encode proteins involved in bacteriophage resistance [37,38].

**Table 5:** Genomic location of each of the four putative temperate phages identified by PHAST.

Region	Start (bp) ^1^	End (bp)^1^	First CDS in the region (predicted function)	Last CDS in the region (predicted function)
1	492404	531549	PVACC_02305 (integrase)	PVACC_02540 (hypothetical protein)
2	1086971	1121038	PVACC_05090 (hypothetical protein)	PVACC_05240 (protease regulator protein HflK)
3	1575333	1619417	PVACC_07385 (ATP-dependent DNA helicase Rep)	PVACC_07675 (cysteine methyltransferase)
4	2223275	2239629	PVACC_10715 (preprotein translocase)	PVACC_10810 (Minor tail protein U)

^1^Genome numbering is relative to the PVAcc strain

### Phage identification

All of the genomes from known HS-associated strains were analysed for the presence of phage elements using PHAST [28]. This analysis identified four regions corresponding to putative temperate phage elements. The genomic locations of these four regions in PVAcc strain, as an example, are presented in Table 5. These regions correspond with the main genetic differences identified between different groups of strains (regions 1–4; Fig 3). Regions 3 and 4 were identified as intact phages and have been reported previously [13]. Region 2 was identified as an incomplete phage and region 1 as a questionable phage (Fig 3). The questionable phage identified in region 1 is present in the seven Pakistani strains, the incomplete phage identified in region 2 is shared by all the Asian strains (relative position BUKK_04735–04880) and the intact phages identified in region 3 and 4 are shared by all the HS strains (relative position BUKK_07250–07540). The phage elements identified in regions 1, 2, 3 and 4 are situated at tRNA^Leu^, tRNA^Met^, tRNA^Ser^ and tRNA^Met^ genes respectively. This correlates with the previous reports that the F108 phage and the lysogenic phage carrying the PMT toxin, integrate into the t33tRNA^Leu^ and the t3tRNA^Leu^ genes respectively [13,40]. Temperate phages may contain important virulence genes [41]. Indeed, as noted above the *P*. *multocida* PMT toxin is the primary virulence factor for porcine atrophic rhinitis and is carried on a lysogenic bacteriophage [42]. Therefore, the presence of different phage elements in different sets of strains may impact on the virulence of these strains. Further studies should assess the impact of these different gene sets on virulence.

A *P*. *multocida* HS-specific diagnostic PCR has been developed previously [15]. We searched for the DNA sequence recognized by this PCR in all genomes and identified it within the putative intact prophage within region 3 (Fig 3), Thus, this sequence is indeed present in all of the HS strains analysed in this study and is not present in any of the non HS-associated genomes analysed.

### Identification of antibiotic resistance genes and characterization of ICE*Pmu2*

The 13 HS-associated genomes were analysed for the presence of acquired antimicrobial resistance genes using the ResFinder tool [29]. It should be noted that ResFinder searches only for acquired resistance genes and not for mutations in chromosomally-encoded genes that lead to antibiotic resistance. Acquired antimicrobial resistance genes were identified only in the BUKK and Tx1 Pakistani isolates. These included three aminoglycoside resistance genes (*strA*, *strB* and *aph(3’)-lc*), one beta lactamase gene (*bla*_TEM-1B_), one chloramphenicol resistance gene (*catA2*), one sulphonamide resistance gene (*sul2*) and one tetracycline resistance gene (*tet(H)*). Thus, these two strains should be resistant to streptomycin, kanamycin/neomycin, beta-lactams, chloramphenicol, sulphonamides and tetracycline. Indeed, this correlates with the clinical data on these isolates as the use of beta lactam antibiotics for infections involving BUKK and TX1 strains has been avoided due to the beta lactamase activity exhibited by these strains (E. Nawaz, personal communication, June, 2012).

All of the identified antibiotic resistance genes were clustered in two regions (relative position BUKK_06700–06910 and 11175–11375). However, it is likely that these regions are colinear within each genome but, due to contig breaks, have been separated in each draft genome assembly. We propose that this region encodes an ICE as many of the genes in this region encode proteins with shared identity to those in the ICE*Pmu1* of strain 36950 [27]. Using 50% identity and 50% gene coverage as the minimum criteria for a match, a total of 77 genes were identified in this region, all of which were shared by the BUKK and TX1 HS strains. Of these, 35 encoded proteins that shared a significant level of identity with proteins in the ICE*Pmu1* region of the multi-drug resistant strain 36950 and a further 16 encoded proteins with lower levels of identity (30 to 50%) to proteins in ICE*Pmu1* (Fig 4). Thus, it is likely that the BUKK and TX1 strains both contain an ICE. The genes with shared identity to the ICE*Pmu1* genes are located in a number of colinear groups within this ICE. These groups are interspersed with the genes identified as unique to strains BUKK and TX1 (Fig 4). The end of one of these colinear groups is flanked by tRNA^Leu^. The equivalent region in strain 36950 represents the right end of the ICE*Pmu1* element (Pmu_03540–03610) and encodes a number of proteins involved in DNA replication, including a single-stranded DNA-binding protein, an ATPase involved in chromosome partitioning, a DnaB-like helicase and a ParB family protein with a predicted DNA nuclease domain. This set of genes has been reported as the most conserved region among diverse proteobacterial ICE [27,43].

Within the putative ICE identified in BUKK and TX1, 47 genes encoded proteins with predicted functions. These included proteins predicted to be involved in ICE mobility, including excision/integration and conjugative transfer. A putative phage integrase was identified (BUKK_06905) with similarity to two integrases found in the ICE*Pmu1* within strain 36950 (identities of 51% and 57% with the first and second integrases, respectively). BUKK_06905 shared significant similarity with tyrosine recombinases of the Xer family, which mediate integration via site-specific recombination. A gene encoding a putative relaxase (BUKK_06900) was identified downstream of this integrase gene; a similar organisation is found in the ICE*Pmu1* [27]. Proteins necessary for conjugative transfer were also present, including proteins predicted to be necessary for the formation of a type IV pilus (BUKK_11185 which shows 53% similarity with Pmu_03230), TraD (BUKK_11205 which shows 73% similarity with Pmu_03190), TraG (BUKK_11275 which shows 60% similarity with Pmu_03040) and TraC (BUKK_11210 which shows 67% similarity with Pmu_03070) [27]. Moreover, a gene encoding a protein with a lysozyme-like domain (BUKK_11195 which shows 52% similarity with Pmu_03210) and a putative DNA topoisomerase III (BUKK_06775) (66% similarity with Pmu_03290 in strain 36950) were also identified.

While the resistance genes *strA* (BUKK_06815) and *strB* (BUKK_06820), *aph(3’)-lc* (BUKK_11370), *sul2* (BUKK_06810) and *tetR*-*tet(H)* (BUKK_11315 and BUKK_11320) are present in strains BUKK, TX1 and 36950 [27,44], the *bla*_TEM-1B_ (BUKK_06875) and *catA2* (BUKK_11355) genes are unique to strains BUKK and TX1. However, strain 36950 contains more resistance genes than strains BUKK and TX1 as it also contains resistance genes for streptomycin/spectinomycin (*aadA25*), gentamicin (*aadB*), kanamycin/neomycin (*aphA1*), chloramphenicol/florfenicol (*floR*), tilmicosin/clindamycin (*erm(42)*) and tilmicosin/tulathromycin (*msr(E)*-*mph(E)*) [44]. These differences indicate that the putative ICE present in strains BUKK and TX1 is not identical to ICE*Pmu1* in strain 36950 and should therefore be designated ICE*Pmu2* as the second ICE discovered in *P*. *multocida*. Further work should aim to close the contig breaks in this region to confirm this is a single element. In addition it would be of great interest to investigate the mobility of ICE*Pmu2*.

## Conclusion

In conclusion, we have shown that HS-associated *P*. *multocida* strains belonging to capsular serogroup B form a very closely related group, but are distinguishable using whole genome analyses. We identified 96 genes unique to the HS-associated strains and future characterization of these genes should elucidate the roles they play in disease pathogenesis, virulence and host specificity. Selected genes from this group will be excellent candidates for the development of a rapid diagnostic test for HS. The putative integrative conjugative element (ICE) identified in two Pakistani isolates should be further analysed to determine its mobility and relatedness to ICE*Pmu1* of strain 36950.

## Supporting Information

S1 File Nucleotide sequence of the strain BUKK capsule biosynthesis locus in FASTA format. (TXT)

S2 File Nucleotide sequence of the strain BUKK LPS biosynthesis locus in FASTA format. (TXT)

S1 Table Genes unique to the HS strains, TX1 strain, Karachi strain, BUKK strain and PVacc strain. (XLSX)
